# Learning-Based IRS-Assisted Secure Transmission for Mine IoTs

**DOI:** 10.3390/s23146321

**Published:** 2023-07-12

**Authors:** Minghui Min, Jiayang Xiao, Peng Zhang, Jinling Song, Shiyin Li

**Affiliations:** 1 School of Information and Control Engineering, China University of Mining and Technology, Xuzhou 221116, China; minmh@cumt.edu.cn (M.M.); xjy2807175506@gmail.com (J.X.);; 2 Key Laboratory of Aerospace Information Security and Trusted Computing, Ministry of Education and School of Cyber Science and Engineering, Wuhan University, Wuhan 430072, China

**Keywords:** Internet of things, mining, active eavesdropping, intelligent reflecting surface, reinforcement learning

## Abstract

Mine Internet of Things (MIoT) devices in intelligent mines often face substantial signal attenuation due to challenging operating conditions. The openness of wireless communication also makes it susceptible to smart attackers, such as active eavesdroppers. The attackers can disrupt equipment operations, compromise production safety, and exfiltrate sensitive environmental data. To address these challenges, we propose an intelligent reflecting surface (IRS)-assisted secure transmission system for an MIoT device which enhances the security and reliability of wireless communication in challenging mining environments. We develop a joint optimization problem for the IRS phase shifts and transmit power, with the goal of enhancing legitimate transmission while suppressing eavesdropping. To accommodate time-varying channel conditions, we propose a reinforcement learning (RL)-based IRS-assisted secure transmission scheme that enables MIoT device to optimize both the IRS reflecting coefficients and transmit power for optimal transmission policy in dynamic environments. We adopt the deep deterministic policy gradient (DDPG) algorithm to explore the optimal transmission policy in continuous space. This can reduce the discretization error caused by traditional RL methods. The simulation results indicate that our proposed scheme achieves superior system utility compared with both the IRS-free (IF) scheme and the IRS randomly configured (IRC) scheme. These results demonstrate the effectiveness and practical relevance of our contributions, proving that implementing IRS in MIoT wireless communication can enhance safety, security, and efficiency in the mining industry.

## 1. Introduction

Mine Internet of Things (MIoT) devices are widely applied in intelligent mines to improve safety and mineral production [1]. In the mining industry, IoT networks play a crucial role in controlling mining equipment and gathering essential environmental data, including temperature, humidity, and wind speed, which are instrumental in safeguarding the personal safety of mine workers [2,3]. The accurate, reliable, and durable operation of MIoT devices is essential for the stable and long-term service of intelligent mines. Therefore, MIoT devices must provide high-speed transmission and low energy consumption. However, the wireless transmission characteristics of electromagnetic waves in MIoT often experience severe scattering, substantial interference, and Non-Line-of-Sight (NLoS) propagation, which necessitates innovation in new transmission technology [4].

Furthermore, despite significant advancements in wireless communication technology in recent decades, most MIoT networks, particularly those deployed in open pit mines, remain susceptible to physical layer threats. The open nature of wireless channels exposes these MIoT devices to vulnerabilities such as jamming and eavesdropping, highlighting the need for enhanced security measures [5]. Malicious devices connected to the system can wiretap confidential information, which can lead to data leakages, such as mineral production schedules, the distribution of mineral resources, and safety aspects of the operations. The intruder can use the stolen data to commit fraud and extortion for illegal profit or pose security threats, such as negative impacts on production and deliberately creating catastrophes. In this case, MIoT devices must be able to withstand smart attacks, particularly active eavesdropping, which involves simultaneous eavesdropping and jamming to increase the MIoT device’s transmit power and intercept more data [6].

As an emerging technology, intelligent reflecting surfaces (IRS) have attracted extensive research interest. The low cost of IRS makes them a highly suitable technology for wide adoption in MIoT communication. IRS contain metamaterial designed to reflect the incident waves from the source towards the destination [7,8]. With properly adjusted elements, IRS can construct an artificial Line-of-Sight (LoS) link and significantly improve transmission performance in NLoS scenes. Moreover, adding the nonreflected signal and the IRS-reflected signal at the eavesdropper can produce destructive interference, effectively suppressing eavesdropping activity [9]. In this paper, IRS establish a favorable propagation environment, increasing the access point (AP)’s received signal power and decreasing the eavesdropper’s received signal power, thus increasing the secrecy rate of the MIoT system in the presence of active eavesdropping.

Due to the complex and random time-varying channel characteristics in MIoT, acquiring the optimal transmission scheme using traditional techniques is typically not feasible [10]. The wiretap policy is also challenging to estimate, making it harder to find the optimum secure transmission policy. Motivated by the advances in model-free deep reinforcement learning (DRL), we model the secure transmission procedure as a Markov Decision Process (MDP). The increasing computational capability of IoT devices, such as the Qualcomm Snap-dragon 800 [11], makes it possible to apply DRL techniques in practical mining IoT communication systems.

In this paper, we propose an innovative secure transmission scheme that leverages IRS and the deep deterministic policy gradient (DDPG) algorithm to enhance the secrecy rate of the system in the presence of an active eavesdropper, specifically in a dynamic MIoT environment. In the proposed scheme, RL is utilized to adapt the time-varying channel characteristics and make the optimal choice without knowing the specific transmission model and attack model. The DDPG-based scheme can select policies in a continuous space while avoiding discretization errors. This enables the MIoT device to jointly optimize the IRS phase shifts and the MIoT device’s transmit power in a mine environment. Strategically adjusting the phase shifts and transmission power of IRS, as well as leveraging the utilization of reflected signals, is helpful to enhance the effectiveness of legitimate transmission and ensure a safe mine environment.

According to our simulation results, the proposed DDPG-based IRS-assisted secure transmission (DIST) scheme achieves higher utility than the IRS randomly configured (IRC) scheme and the IRS-free (IF) scheme. By changing the number of IRS elements, we also assess the system utility of both the proposed DIST scheme and the IRC scheme. The main contributions of this paper can be outlined as follows:

- We construct a joint optimization problem of the MIoT device’s transmit power and IRS reflecting beamforming to maximize the system’s utility. We present an IRS-assisted secure transmission scheme against active eavesdropping in MIoT.
- A DRL-based intelligent beamforming and power control framework is presented to achieve the optimal IRS phase shifts and MIoT device’s transmit power. We formulate the control of the IRS elements as an MDP and employ the DDPG algorithm to achieve real-time and continuous phase control based on the dynamic MIoT environment.
- Simulation results demonstrate our proposed DRL-based IRS-assisted secure transmission scheme’s performance suppresses eavesdropping and enhances legitimate transmission compared with the IRC and IF schemes.

The subsequent sections of this paper are organized as follows: Section 2 discusses the related works. Section 3 introduces the proposed system model, channel model, and problem formulation. In Section 4, we introduce our proposed DIST scheme. Section 5 provides simulation results, and Section 6 concludes the paper.

Notations: In this paper, we present matrices and vectors with boldface. ${\left(.\right)}^{T}$ and ${\left(.\right)}^{H}$ denote the transpose and conjugate transpose operations, respectively. $diag\left(.\right)$ denotes a diagonal matrix, and *j* is the imaginary unit. $E\left[.\right]$ denotes the expectation operation. $\left|.\right|$ is the absolute value of a scalar. $C^{M\times N}$ denotes a complex-valued matrix with a size of M×N.

## 2. Related Works

Numerous methods have been proposed to improve physical layer security (PLS) performance, including artificial noise (AN) [12], physical layer authentication (PLA) [13], and beamforming [14]. However, these methods have limitations, such as extra power consumption for AN, computing resource requirements for PLA, and limited security guarantees for beamforming. For mining scenario, the authors in [15] investigate PLS in an underground mine environment using an amplify-and-forward relay-aided system with multiple eavesdroppers. The authors employ a block coordinate descent algorithm to design the precoding and jamming matrix at both the source and the relay, similar to other traditional PLS techniques, rather than during the propagation process. Recently, the use of IRS has gained significant attention to address PLS issues in the propagation process. Several studies have explored the use of IRS in secure communication systems in [16,17,18,19]. A genetic algorithm (GA) is introduced in [16] to optimize the phase shift of an IRS in a multiple-input multiple-output (MIMO) system, with the goal of improving security performance in the presence of an eavesdropper. To reduce the overhead of computing resources, a low-complexity algorithm is studied in [17] based on fractional programming (FP) and manifold optimization (MO) to circumvent the nonconvex optimization problem and obtain near-optimal IRS phase shifts. However, the optimization technique in both [16,17] rely on a specific transmission model and lack robustness. Moreover, a more practical system model comprising multiple eavesdroppers and imperfect channel state information (CSI) are studied in [18,19]. The interuser interference (IUI) among each mobile user (MU) is studied in [19]. Additionally, none of the existing IRS-assisted PLS approaches consider an active eavesdropper scenario where jamming attacks interfere with the legitimate transmission and raise the transmit power.

Artificial Intelligence (AI) has introduced a new way to solve PLS problems through RL. Recent studies in [20,21] have considered PLS problems concerning smart attackers conducting jamming, eavesdropping, and spoofing attacks. For instance, prospect theory (PT) in an unmanned aerial vehicle (UAV) transmission system is investigated in [20], where the attacker is considered to be selfish and subjective. To enhance the secrecy performance and the utility of the legitimate UAV, a power allocation approach utilizing deep Q-networks (DQN) is put forth to determine the optimal policy, in cases where the attack and channel models are unknown. RL techniques are studied in [21] to configure IRS beamforming design. The authors first establish the interaction between the base station (BS) and the smart attacker as a non-cooperative game and derive the Nash equilibrium of the game. Then, a DQN-based antismart attacker strategy is proposed to make the BS and IRS intelligent and restrain the attack, thus improving the system’s security. However, since the study assumes a static channel, the proposed strategy may be less adaptable to varying channel conditions, despite its focus on the game-theoretic interaction between the base station (BS) and the attacker. To address these limitations, a novel DRL framework is proposed in [22] to enable the prediction of IRS reflection matrices without the need for extensive channel estimation or beamforming train overhead. Additionally, an integrated DRL and extremum-seeking control (ESC) is studied in [23] to control the IRS and make the system more adaptive to the dynamic channel state without subchannel CSI.

The implementation of IRS and RL in the mining industry is a relatively unexplored research area. Machine learning is applied in a mining system to remove the operator from hazardous environments without compromising task execution [24]. Ref. [25] is the first work implementing IRS in a coal mine. In this study, IRS are placed at the inflection points of the nonlinear routes (i.e., zigzag tunnels) to improve wireless communication quality. Although an approximation-based algorithm is utilized to address the optimization problem, the complex and dynamic nature of the channel state is ignored. Thus, the proposed method in [25] may not be practical in most mining scenarios. Furthermore, neither [24] nor [25] use RL or IRS to solve the PLS problems and enhance secure transmission in a mine environment. The mainly related work is summarized in Table 1.

## 3. System Model and Problem Formulation

### 3.1. System Model

**Figure 1:**
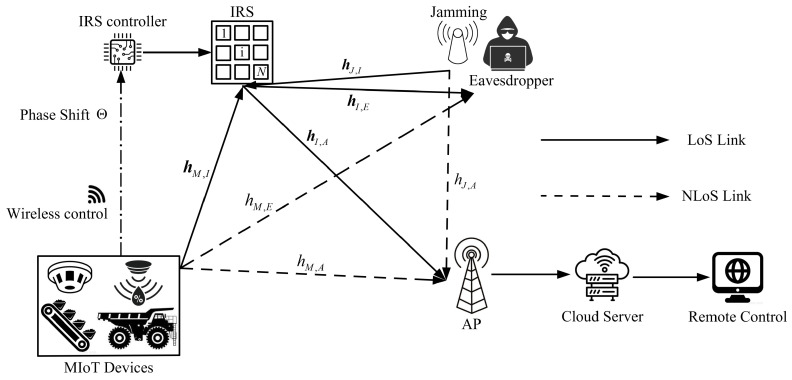
Illustration of the IRS-assisted secure transmission system in MIoT. The MIoT devices choose the transmit power and send phase shift control messages to the IRS controller. At the same time, the active eavesdropper performs jamming to increase the MIoT devices’ transmit power for a higher wiretap rate.

Considering a single-input single-output (SISO) uplink system, as shown in Figure 1, one MIoT device, equipped with a single antenna, establishes communication with a single-antenna AP. Simultaneously, we introduce a single-antenna active eavesdropper with the intention of intercepting the transmission. The MIoT device collects data, such as temperature and gas density, and transmits the data to the AP, which is located $d_{M,A}$ meters away. To establish a dependable communication environment, a passive IRS is deployed at a distance of $d_{M,I}$ meters from the MIoT device, with $N=N_{y}\times N_{z}$ reflecting elements. All elements are configured through a wireless IRS controller that receives the control signal from the MIoT device. The IRS reflect the signal to enhance the transmission from the MIoT device to the AP and suppress the wiretap signal at the eavesdropper, thereby obtaining the maximum secrecy rate. The data are then updated to a cloud server and used by the remote control on the ground for digital management in the mine IoT applications.

Upon receiving the control signal, the micro IRS controller sets the bias voltage to apply the phase shift on each IRS reflecting element. The phase shifts configuration can be modeled as $\Theta =diag\left(\beta_{1}e^{j\theta_{1}},\beta_{2}e^{j\theta_{2}},\ldots ,\beta_{N}e^{j\theta_{N}}\right)$, where $\beta_{N}\in \left[0,1\right]$ and $\theta_{N}\in \left[0,2\pi \right)$ are the amplitude reflection coefficient and phase shift of the *n*-th IRS element, respectively. For simplicity, we set $\beta_{N}=1$ for *N* reflecting elements.

The transmission policy in an IRS-assisted secure transmission system relies on precisely acquiring CSI. In our proposed system model, the legitimate channel state is obtained by the pilot-based channel estimation [26]. We also assume the CSI of the wiretap channel to be perfectly known to the MIoT device. This is because the eavesdropper is considered an active user in the system but is not trusted by the legitimate receiver [9].

### 3.2. Channel Model

The channel path losses from the MIoT device to the AP, from the MIoT to the eavesdropper, and from the jamming to the AP are denoted by $h_{M,A}$, $h_{M,E}$, and $h_{J,A}$. The channel path losses above are all regarded as Rayleigh fading, which means that the Line-of-Sight signal between the transmitter and receiver is blocked and can be expressed as [27]:

(1) $$\begin{array}{c} h_{M,A}=\sqrt{PL_{M,A}}{\tilde{h}}_{M,A}\\ h_{M,E}=\sqrt{PL_{M,E}}{\tilde{h}}_{M,E}\\ h_{J,A}=\sqrt{PL_{J,A}}{\tilde{h}}_{J,A} \end{array}$$

where PL is the path loss. $\tilde{h}$ contains independent and identically distributed (i.i.d) circularly symmetric complex Gaussian distribution with zero mean and unit variance, $\tilde{h}∼\mathrm{CN}\left(0,1\right)$.

The distance-dependent path loss PL is modeled as

(2) $$PL=PL_{0}-10\xi \log_{10}\frac{d}{d_{0}}$$

where $PL_{0}=-30$ dB is the reference channel path loss for the reference distance $d_{0}=1$ m, ξ is the path loss exponent, and *d* is the distance from the transmitter to the receiver.

The channel path loss from the MIoT device to the IRS, from the IRS to the AP, from the IRS to the eavesdropper, and from the jamming to the IRS are denoted by $h_{M,I}\in C^{N\times 1}$, $h_{I,A}\in C^{N\times 1}$, $h_{I,E}\in C^{N\times 1}$, and $h_{J,I}\in C^{N\times 1}$. The channel path losses above are all assumed to be small-scale Rician fading, which suggests the LoS link coexists with NLoS link, and the channel path loss can be expressed as [7,23]

(3) $$\begin{array}{c} h_{M,I}=\sqrt{PL_{M,I}}\left(\sqrt{\frac{K_{M,I}}{K_{M,I}+1}}{\bar{h}}_{M,I}+\sqrt{\frac{1}{K_{M,I}+1}}{\tilde{h}}_{M,I}\right)\\ h_{I,A}=\sqrt{PL_{I,A}}\left(\sqrt{\frac{K_{I,A}}{K_{I,A}+1}}{\bar{h}}_{I,A}+\sqrt{\frac{1}{K_{I,A}+1}}{\tilde{h}}_{I,A}\right)\\ h_{I,E}=\sqrt{PL_{I,E}}\left(\sqrt{\frac{K_{I,E}}{K_{I,E}+1}}{\bar{h}}_{I,E}+\sqrt{\frac{1}{K_{I,E}+1}}{\tilde{h}}_{I,E}\right)\\ h_{J,I}=\sqrt{PL_{J,I}}\left(\sqrt{\frac{K_{J,I}}{K_{J,I}+1}}{\bar{h}}_{J,I}+\sqrt{\frac{1}{K_{J,I}+1}}{\tilde{h}}_{J,I}\right) \end{array}$$

where *K* is the Rician-K factor and denotes the proportion of power between the LoS link and the NLoS link. $\tilde{h}$ is the random components caused by multipath effect with i.i.d and CN(0,1) distributed elements. The deterministic component $\bar{h}$ is position-dependent and can be expressed as [28]

(4) $$\bar{h}=h^{A}h^{D}$$

where the superscripts “A” and “D” stand for “Arrival” and “Departure”, respectively.

Without loss of generality, we place the IRS on the yOz plane. So, the component $h^{A\left(D\right)}$ in Equation (4) can be expressed as

(5) $$h^{A\left(D\right)}=h_{y-axis}^{A\left(D\right)}h_{z-axis}^{A\left(D\right)}$$

where

(6) $$h_{z-axis}^{A\left(D\right)}={\left[1,e^{-j\frac{2\pi }{\lambda_{c}}d\cos\psi },\ldots ,e^{-j\frac{2\pi }{\lambda_{c}}d\cos\vartheta \left(N_{z}-1\right)}\right]}^{T}$$

(7) $$h_{y-axis}^{A\left(D\right)}=\left[1,e^{-j\frac{2\pi }{\lambda_{c}}d\cos\vartheta \sin\psi },\ldots ,e^{-j\frac{2\pi }{\lambda_{c}}d\cos\vartheta \sin\psi \left(N_{y}-1\right)}\right]$$

where $\lambda_{c}$ is the carrier wavelength, and *d* is the distance between two adjacent IRS elements. Furthermore, ϑ represents the azimuth angle and ψ represents the elevation angle. The LoS component $\bar{h}$ is solely dependent on ϑ and ψ, meaning that once the locations of each unit are obtained, $\bar{h}$ is fully determined.

For the proposed system model, the MIoT device sends message *m* with zero mean and unit variance to the AP with transmission power *p*, where $E\left\{{\left|m\right|}^{2}\right\}=1$, $p\in \left[P_{\min},P_{\max}\right]$, $P_{\min}$, and $P_{\max}$ is the minimum, and the maximum values of the MIoT device transmit power, respectively.

### 3.3. Problem Formulation

The received signal $y_{A}$ at the AP and $y_{E}$ [9] at the eavesdropper can be denoted as

(8) $$y_{A}=\left(h_{I,A}^{H}\Theta h_{M,I}+h_{M,A}\right)\sqrt{p}m+n_{k}$$

(9) $$y_{E}=\left(h_{I,E}^{H}\Theta h_{M,I}+h_{M,E}\right)\sqrt{p}m+n_{k}$$

where $n_{k}∼\mathrm{CN}\left(0,\sigma^{2}\right)$ denotes the complex additive white Gaussian noise (AWGN).

The active eavesdropper aims to wiretap more data by increasing the jamming power $p_{J}$. Therefore, we assume that the active eavesdropper has no self-interference. That is to say, we ignore the LoS channel between the eavesdropper and the jamming device and only consider the IRS-reflected jamming signals. Thus, the received jamming signal $J_{A}$ at the AP and $J_{E}$ at the eavesdropper can be expressed as

(10) $$J_{A}=\left(h_{I,A}^{H}\Theta h_{J,I}+h_{J,A}\right)\sqrt{p_{J}}+n_{k}$$

(11) $$J_{E}=\left(h_{I,E}^{H}\Theta h_{J,I}\right)\sqrt{p_{J}}+n_{k}$$

Then, we can calculate the signal-to-interference-and-noise ratio (SINR) $\rho_{A}$ at the AP and $\rho_{E}$ at the eavesdropper [29], and they can be expressed as

(12) $$\rho_{A}=\frac{{\left|\left(h_{I,A}^{H}\Theta h_{M,I}+h_{M,A}\right)\sqrt{p}\right|}^{2}}{{\left|\left(h_{I,A}^{H}\Theta h_{J,I}+h_{J,A}\right)\sqrt{p_{J}}\right|}^{2}+\sigma^{2}}$$

(13) $$\rho_{E}=\frac{{\left|\left(h_{I,E}^{H}\Theta h_{M,I}+h_{M,E}\right)\sqrt{p}\right|}^{2}}{{\left|\left(h_{I,E}^{H}\Theta h_{J,I}\right)\sqrt{p_{J}}\right|}^{2}+\sigma^{2}}$$

We evaluate the eavesdropping policy according to the AP’s received jamming power ${\tilde{p}}_{J}$, which can be denoted as

(14) $${\tilde{p}}_{J}={\left|\left(h_{I,A}^{H}\Theta h_{J,I}+h_{J,A}\right)\sqrt{p_{J}}\right|}^{2}$$

The achievable rates at AP $R_{A}$ and eavesdropper $R_{E}$ in bps/Hz can be denoted as [6,19]

(15) $$R_{A}=\log_{2}\left(1+\rho_{A}\right)$$

(16) $$R_{E}=\log_{2}\left(1+\rho_{E}\right)$$

Thus, the achievable secrecy rate $R_{sec}$ [19,30] can be denoted as

(17) $$R_{sec}=R_{A}-R_{E}$$

To achieve the maximum secrecy rate $R_{sec}$, there is a trade-off in configuring the IRS reflecting coefficient matrix Θ. On the one hand, we synchronize the phase of the reflected channel $h_{I,A}^{H}\Theta h_{M,I}$ with the direct channel $h_{M,A}$ to strengthen AP’s received signal and thus maximize $R_{A}$. On the other hand, we reverse synchronize the phase of the reflect channel $h_{I,E}^{H}\Theta h_{M,I}$ with the direct channel $h_{M,E}$ to weaken the eavesdropper’s received signal and decrease $R_{E}$.

Then, the MIoT system’s utility function [6] is defined as follows:

(18) $$U\left(\theta ,p\right)=\omega_{1}R_{sec}-\omega_{2}p$$

where $\theta =[\theta_{1},\ldots ,\theta_{n},\ldots ,\theta_{N}]$, $\forall n\in \left\{1,2,\ldots ,N\right\}$; weights $\omega_{1}$ and $\omega_{2}$ denote the coefficients. The coefficients $\omega_{1}$ and $\omega_{2}$ represent the weight of the achievable secrecy rate and the transmit power, which are set for balancing the influence factors of the utility function.

We aim to optimize the IRS phase shifts θ and the MIoT device’s transmit power *p* to maximize the utility. The following formulation represents the optimization problem:

(19) $$\begin{array}{cc} \; & \max_{\theta ,p}\;U\left(\theta ,p\right)\\ \; & s.t.\;\left\{\begin{array}{c} \theta_{n}\in \left[0,2\pi \right)\\ p\in \left[p_{\min},p_{\max}\right] \end{array}\right. \end{array}$$

However, it is difficult to solve the formulated problem, as its objective function is nonconvex concerning either θ or *p*. Additionally, the complex time-varying channel fading makes it impossible to obtain an optimal solution for long-term system utility using traditional optimization techniques.

## 4. Proposed DIST Scheme

### 4.1. Main Elements of DIST

In previous sections, we discussed the challenges in MIoT wireless communication. To address these issues, we propose a model-free RL approach. More specifically, we introduce a DDPG-based IRS-assisted secure transmission (DIST) scheme to efficiently search the policy space and improve the secure transmission performance while remaining independent of any specific system model or wiretap policy [31]. The DIST scheme is designed to be applicable to a wide range of MIoT systems, making it a valuable contribution to the field. By considering the IRS-assisted MIoT device’s transmission system as the dynamic environment and the MIoT device itself as the learning agent, our method is able to adapt to various situations and effectively address the security concerns in MIoT wireless communication. In the following specifications, we outline the main components of the framework employed by the DIST scheme.

**State space:** At time slot *k*, the MIoT device observes the environment and formulates the state $s^{\left(k\right)}$, which is modeled as follows:

(20) $$s^{\left(k\right)}=\left[h^{\left(k\right)},{\tilde{p_{J}}}^{\left(k-1\right)}\right]\in \Lambda$$

where Λ is the state space. $h^{\left(k\right)}=\left\{h_{M,I}^{\left(k\right)},h_{M,A}^{\left(k\right)},h_{M,E}^{\left(k\right)},h_{I,A}^{\left(k\right)},h_{J,I}^{\left(k\right)},h_{I,E}^{\left(k\right)},h_{J,A}^{\left(k\right)}\right\}$, $h^{\left(k\right)}$ are the channel path loss at time slot *k*. ${\tilde{p_{J}}}^{\left(k-1\right)}$ is the AP’s received jamming power at time slot k−1.

**Action space:** We denote A as the action space. According to the observed state $s^{\left(k\right)}$ at time slot *k*, the MIoT device designs the IRS phase shifts $\theta^{\left(k\right)}$ and chooses the transmit power $p^{\left(k\right)}$. Then, the phase shifts control signal is sent to the IRS controller. Hence, the secure transmission policy $a^{\left(k\right)}\in A$ can be formulated by

(21) $$a^{\left(k\right)}=\left[\theta^{\left(k\right)},p^{\left(k\right)}\right]$$

**Reward function:** In the proposed DIST scheme, the reward function evaluates the secure transmission policy according to the current state. In the presented paper, we aim to achieve the maximum long-term utility of the system, as addressed in Equation (19). Thus, the reward function is denoted as follows:

(22) $$r^{\left(k\right)}\left(s,a\right)=U^{\left(k\right)}$$

### 4.2. Main Process of DIST

Our proposed DIST scheme contains a critic network and an actor network, denoted as $Q\left(s,a|\Psi \right)$ and $\mu \left(s|\Omega \right)$ with parameters Ψ and Ω, respectively. The actor network is responsible for choosing the secure transmission policy, while the critic network assesses the policy selected by the actor network. Moreover, a target critic network $Q{}^{\prime }\left(s,a|\Psi {}^{\prime }\right)$ and a target actor network $\mu {}^{\prime }\left(s|\Omega {}^{\prime }\right)$ are designed to promote convergence.

At the beginning of each episode, the MIoT device sets a random phase shift on each element. The MIoT device observes the environment and acquires the global CSI and the AP’s received jamming power. Then, the MIoT device formulates the initial state s and inputs it into the actor network to generate corresponding transmission policy a.

According to the observed state $s^{\left(k\right)}$ at time slot *k*, the MIoT device selects the secure transmission policy $a^{\left(k\right)}=\left[\theta^{\left(k\right)},p^{\left(k\right)}\right]$ through the actor network. The actor network links each state to a corresponding transmission policy using function $\mu \left(s^{\left(k\right)}|\Omega^{\left(k\right)}\right)$. To enable the MIoT device to explore the environment, we model an Ornstein–Uhlenbeck (OU) process as the exploration noise $N^{\left(k\right)}$, which is known as the OU-noise. The OU-noise is used to improve the exploration efficiency and find the optimal policy with better convergence. Thus, the secure transmission policy $a^{\left(k\right)}$ is given by

(23) $$a^{\left(k\right)}=\mu \left(s^{\left(k\right)}|\Omega^{\left(k\right)}\right)+N^{\left(k\right)}$$

**Figure 2:**
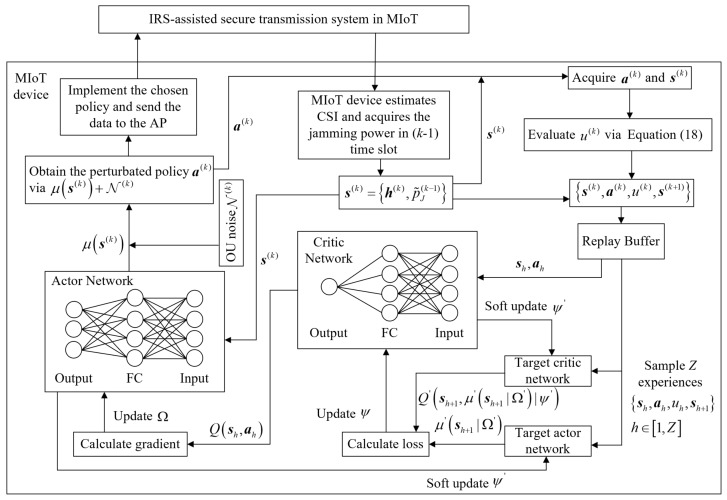
The DDPG-based IRS-assisted secure transmission scheme in MIoT.

The MIoT device then sends the phase shifts control signal to the IRS controller and transmits the data to the AP with the transmit power *p*. Then, the MIoT device calculates the achievable rate at AP and eavesdropper via Equations (15) and (16). As a result, the MIoT obtains an immediate reward $u^{\left(k\right)}$, and the system state $s^{\left(k\right)}$ is updated to a new state $s^{\left(k+1\right)}$, which is denoted as $s^{\left(k+1\right)}=\left[h^{\left(k+1\right)},{\tilde{p_{J}}}^{\left(k\right)}\right]$. Next, the MIoT device stores the transition $\left(s^{\left(k\right)},a^{\left(k\right)},u^{\left(k\right)},s^{\left(k+1\right)}\right)$ in the replay buffer, where the oldest experience is systematically discarded in a rolling manner as the buffer reaches its maximum capacity. When the buffer size is larger than the batch size *Z*, the MIoT device randomly samples *Z* experiences from the replay buffer for exploring the optimal transmission policy in the dynamic MIoT environment. The detailed structure is shown in Figure 2.

We formulate the minibatch $e_{h}=\left\{s_{h},a_{h},u_{h},s_{h+1}\right\}$, $h\in \left[1,Z\right]$ and utilize the Adam optimizer to update the critic network’s weight Ψ [32], where the loss function is denoted as

(24) $$\Psi =\arg\;\min_{\Psi }\frac{1}{H}\sum_{h=1}^{H}{\left(u_{h}+\gamma Q{}^{\prime }\left(s_{h+1},\mu {}^{\prime }\left(s_{h+1}|\Omega {}^{\prime }\right)|\Psi {}^{\prime }\right)-Q\left(s_{h},a_{h}|\Psi \right)\right)}^{2}$$

where the discount factor $\gamma \in \left[0,1\right]$.

The weights of the actor network are updated by leveraging the gradient of the Q-value [32], which can be expressed as follows:

(25) $${▽}_{\Omega }\approx \frac{1}{H}\sum_{h=1}^{H}{▽}_{a}Q\left(s=s_{h},a=\mu \left(s_{h}\right)|\Psi \right)\times {▽}_{\Omega }\mu \left(s=s_{h}|\Omega \right)$$

Lastly, the MIoT device uses the soft update strategy to ensure the target network changes slowly, thus guaranteeing stability. The soft update can be denoted as follows:

(26) $$\begin{array}{cc} \; & \Psi {}^{\prime }=\tau \Psi +\left(1-\tau \right)\Psi {}^{\prime }\\ \; & \Omega {}^{\prime }=\tau \Omega +\left(1-\tau \right)\Omega {}^{\prime } \end{array}$$

where the τ represents the learning rate. The more detailed process is illustrated in Algorithm 1.
**Algorithm 1** DDPG-based IRS-assisted secure transmission scheme (DIST)**Initialize:** actor network, critic network, target critic network, target actor network, and replay buffer1:**for** episode e = 1, 2, 3, …, E **do**2:    Initialize action exploration noise N3:    Obtain the channel state information $h_{M,I}$, $h_{M,A}$, $h_{M,E}$, $h_{I,A}$, $h_{J,I}$, $h_{I,E}$, $h_{J,A}$4:    Randomly choose the IRS phase shifts θ5:    Evaluate the AP’s received jamming power $\tilde{p_{J}}$6:    Formulate the initial state *s* according to Equation (20)7:    **for** Time slot k = 1, 2, 3, …, T **do**8:          Select transmission policy $a^{\left(k\right)}$ with state $s^{\left(k\right)}$ and noise $N^{\left(k\right)}$ based on the current policy.9:          Execute transmission policy $a^{\left(k\right)}$ and obtain the reward and utility $U^{\left(k\right)}=r^{\left(k\right)}\left(s,a\right)$10:        Obtain the AP’s received jamming power ${\tilde{p_{J}}}^{\left(k\right)}$11:        Obtain the channel path loss $h_{M,I}^{\left(k+1\right)}$, $h_{M,A}^{\left(k+1\right)}$, $h_{M,E}^{\left(k+1\right)}$, $h_{I,A}^{\left(k+1\right)}$, $h_{J,I}^{\left(k+1\right)}$, $h_{I,E}^{\left(k+1\right)}$, $h_{J,A}^{\left(k+1\right)}$12:        Formulate the state $s^{\left(k+1\right)}$13:        Store the transition $\left(s^{\left(k\right)},a^{\left(k\right)},u^{\left(k\right)},s^{\left(k+1\right)}\right)$ to the replay buffer14:        **if** buffer length > *Z* **then**15:           Randomly sample a minibatch of *Z* transitions $\left(s_{h},a_{h},u_{h},s_{h+1}\right)$16:           Update the critic network and actor network via Equations (24) and (25)17:           Update the target actor network and target critic network via Equation (26)18:        **end if**19:    **end for**20:**end for**

In Table 2, we present the advantages and potential limitations associated with the DIST scheme.

## 5. Simulation Setup and Results

**Figure 3:**
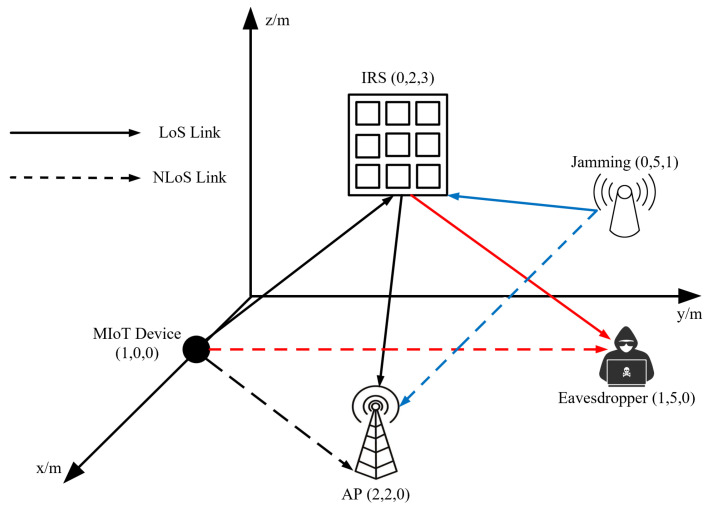
Simulation setting for an IRS-assisted secure transmission system in MIoT.

In this section, we comprehensively illustrate the performance of our proposed DIST scheme under the presence of an active eavesdropper in mining scenarios. The system topology and coordinate of each unit are shown in Figure 3. The red line, blue line, and black line represent the eavesdropping channel, jamming channel, and legitimate transmission channel, respectively. In real-world mining operations, the positions of devices may vary. The changing positions may affect the value of the system performance. However, it will not impact the advantage trend of the proposed DIST scheme compared with the benchmarks. Simulations are implemented using Pytorch 1.13.1 with Python 3.9. The number of MIoT devices, jamming devices, and active eavesdroppers is set to 1, and they are all equipped with one single antenna. The MIoT device observes and estimates the CSI at each time slot. The IRS is composed of a total of N=12 [6] reflecting elements, specifically $N_{y}=2$ elements aligned parallel to the y-axis and $N_{z}=6$ elements aligned parallel to the z-axis. The background noise power $\sigma^{2}$ is set to −80 dBm [27]. The MIoT device is specifically configured to operate within a transmit power range, with a minimum power $P_{\min}$ setting of 1 mW and a maximum power $P_{\max}$ setting of 9 mW. The jamming power is randomly generated in the range of 1 mW and 5 mW. The Rician factors $K_{M,I}$, $K_{I,A}$, $K_{I,E}$, and $K_{J,I}$ are assumed to be equal and set to 10 [33]. $\xi_{LoS}=2.2$ and $\xi_{NLoS}=3.8$ [34] are the path loss exponents of the LoS link and NLoS link, respectively.

The learning model in the proposed DIST framework consists of a three-layer deep neural network (DNN). The hidden layer contains 32 neurons. The actor and critic learning rates are set to $5\times 10^{-7}$ and $5\times 10^{-4}$, respectively. Moreover, the discount factor is determined to be γ=0.3, whereas the soft update parameter is configured to be τ=0.005. We set the max buffer size to 10,240 and the batch size to 16. Moreover, we set the time slot number in each episode to T=256 and the episode number to E=1024. The parameters $\omega_{1}$ and $\omega_{2}$ in Equation (18) are set to 1 and 500, respectively, to balance the secrecy rate gain and power consumption loss. For the settings of the parameters mentioned above, we determined them through multiple experiments conducted by our research team.

Two benchmark schemes are considered, shown as follows:

**IRS randomly configured (IRC)**: In this case, the reflection coefficients of each IRS element are generated randomly. We only use the DDPG algorithm to optimize the transmit power [35].

**IRS-free (IF)**: We consider a classical communication system in MIoT without introducing the IRS. In this case, the MIoT device only chooses the transmit power based on the DDPG algorithm [36].

**Figure 4:**
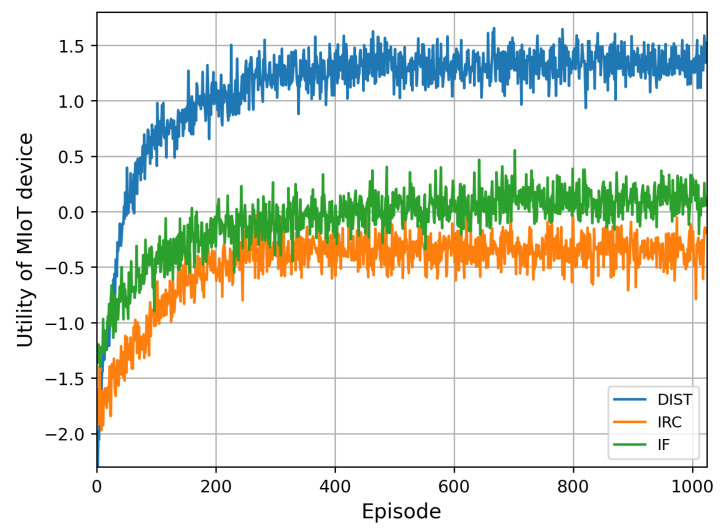
Utility of MIoT device of the DIST scheme compared with the IRC and IF schemes.

Figure 4 provides a comprehensive evaluation of the system utility across all schemes. Our proposed DIST scheme converges after 400 episodes and achieves the utility increment from −1.8 to 1.3. Specifically, in episode 600, our proposed DIST scheme achieves 5.5 and 4.25 times higher utility than the IF and IRC schemes, respectively. This proves the remarkable utility increase from applying the IRS in MIoT wireless communication. And it also emphasizes the significance of applying the RL mechanism to solve the IRS beamforming design problem in a secure transmission scene.

**Figure 5:**
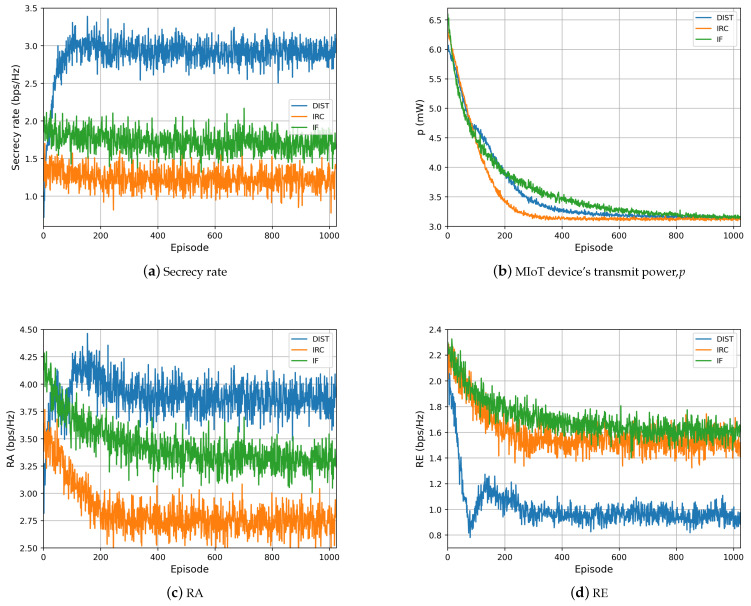
Performance of our proposed DIST scheme compared with the IRC and IF schemes: (**a**) Secrecy rate, $R_{sec}$. (**b**) MIoT device’s transmit power, *p*. (**c**) Signal transmission rate at AP, $R_{A}$. (**d**) Eavesdropping rate, $R_{E}$.

Figure 5 investigates the $R_{A}$, $R_{E}$, $R_{sec}$, and *p* of all schemes. For the secrecy rate shown in Figure 5a, our proposed DIST scheme outperforms the IF scheme and the IRC scheme by 70.6% and 141.7% in secrecy capacity. We then dig into the detailed performance. Particularly, in Figure 5d, in our proposed DIST scheme, we observe that the eavesdropping rate increases from 0.8 bps/Hz to 1.2 bps/Hz from episode 80 to 160, and then falls to 0.9 bps/Hz. The reason is that the MIoT device explores the environment and chooses the policy aiming to obtain the maximum utility. In this process, the eavesdropping rate may go up a bit, but in Figure 5a,c, the signal transmission rate at AP and the secrecy rate are still rising. Several factors contribute to the continuous rise in system utility in this interval. Among these are the factors mentioned above and the declining transmit power, as shown in Figure 5b.

Additionally, in Figure 5c, the signal transmission rate at AP degrades a little bit from 4.25 bps/Hz to 3.9 bps/Hz after 190 episodes. The reason is that the MIoT device’s transmit power converges more slowly than the IRS phase shifts. After 200 episodes, the transmit power is still declining. According to Equations (12) and (15), lower transmit power will lead to a lower AP signal transmission rate when the reflecting coefficients converge to the optimal value.

**Figure 6:**
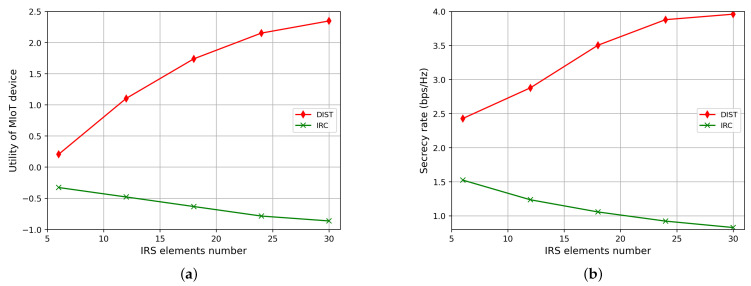
Average performance of the DIST scheme and the IRC scheme in MIoT, with the IRS elements number changing from 6 to 30 and averaged over 1024 episodes against active eavesdroppers: (**a**) Utility of MIoT device. (**b**) Secrecy rate.

As shown in Figure 6, we investigate the performance of our proposed DIST scheme and the IRC scheme by varying the number of IRS elements. The significant improvement of our proposed scheme demonstrated in Figure 6 results from more IRS elements bringing more reflected signals. When the IRS are well-adjusted, the reflected signal can be intelligently combined at AP to provide higher signal strength and deliberately manipulated at the eavesdropper to attenuate its received signal power, thereby diminishing its ability to intercept the transmission.

Moreover, the system utility of the IRC scheme decreases slightly as the number of IRS elements increases. This is because without IRS properly adjusted, the reflected signal with random phase will be added constructively or destructively, generating a stronger or weaker signal. Thus, the more IRS elements used, the larger the range of the SINR. According to Equations (15) and (16), the average $R_{A}$ and $R_{E}$ will decrease due to the different slope of function $\log_{2}\left(x\right)$ when the SINR range gets bigger, eventually resulting in performance degradation.

## 6. Conclusions

In this paper, we investigated a secure transmission scheme against an active eavesdropper and formulated the optimization problem to maximize the utility of an MIoT device for a dynamic MIoT communication environment. We proposed a DDPG-based IRS-assisted secure transmission scheme in MIoT that enables the MIoT device to jointly design the optimal IRS phase shifts and transmit power. Simulation results demonstrate the effectiveness of our proposed scheme in enhancing secrecy rates and reducing power consumption. Comparing our DIST scheme with the IF scheme and the IRC scheme, our DIST scheme achieves a substantial performance improvement in utility of 5.5 times and 4.25 times, respectively. These results demonstrate the vital role of IRS in bolstering physical layer security and enhancing transmission performance in the MIoT wireless communication environment. This work can also be applied to handle secure transmission in other NLoS scenarios, such as large-scale underground supermarkets. Our future work will focus on developing a multiagent learning-based method to solve multidevice scenarios, including multiple receivers and eavesdroppers.

## Figures and Tables

**Table 1:** Summary of related literature.

Classification	Related Works	Key Contributions
Traditional techniques used to achieve PLS	[12,13,14,15]	- Artificial noise, physical layer authentication, and beamforming techniques. - PLS in underground mine environments with relay-aided systems.
Implementation of IRS in PLS	[16,17,18,19]	- Genetic algorithms for IRS phase shift optimization. - Low-complexity algorithms for IRS optimization. - Researches on practical system models.
Implementation of IRS and RL to achieve PLS	[20,21,22,23]	- Power allocation using deep Q-networks for UAV systems. - IRS beamforming design using RL techniques. - DRL-based adaptive IRS control.
Implementation of IRS and machine learning in mining systems	[24,25]	- Machine learning applications in mining systems. - Implementation of IRS in coal mines.

**Table 2:** Advantages and limitations of the proposed DIST scheme.

Advantages	Limitations
Adapt to time-varying and dynamic channel conditions	In practice, MIoT devices can hardly obtain the perfect CSI in a timely manner, causing performance degradation
Reduce transmit power consumption and promote energy efficiency	Computationally intensive due to the application of DRL
Enhance wireless communication in challenging mining environments	Only suitable for single-device scenarios

## Data Availability

Not applicable.
